# Feasibility Study of a New Magnetic Resonance Imaging Mini-capsule Device to Measure Whole Gut Transit Time in Paediatric Constipation

**DOI:** 10.1097/MPG.0000000000002910

**Published:** 2020-08-17

**Authors:** Hayfa Sharif, Nichola Abrehart, Caroline L. Hoad, Kathryn Murray, Alan C. Perkins, Murray Smith, Penny A. Gowland, Robin C. Spiller, Roy Harris, Sian Kirkham, Sabarinathan Loganathan, Michalis Papadopoulos, Kate Frost, David Devadason, Luca Marciani

**Affiliations:** ∗ Nottingham Digestive Diseases Centre and National Institute for Health Research (NIHR), Nottingham Biomedical Research Centre, Nottingham University Hospitals NHS Trust, University of Nottingham, Nottingham, UK; † Amiri Hospital, Ministry of Health, Civil Service Commission, Kuwait City, Kuwait; ‡ Sir Peter Mansfield Imaging Centre, School of Physics and Astronomy, University of Nottingham, Nottingham; § Radiological Sciences, School of Medicine, University of Nottingham, Nottingham; || School of Health and Social Care, College of Social Science, University of Lincoln, Lincoln; ¶ Nottingham Children's Hospital, Nottingham University Hospitals NHS Trust, Queen's Medical Centre; # Nottingham University Hospitals Young Persons Advisory Group, Nottingham University Hospitals NHS Trust, Nottingham, UK.

**Keywords:** gastrointestinal tract, magnetic resonance imaging, Magnetic Resonance Imaging in Paediatric Constipation, TransiCap mini-capsules, x-ray radiopaque markers

## Abstract

**Objective:**

In England, 27,500 children are referred annually to hospital with constipation. An objective measure of whole gut transit time (WGTT) could aid management. The current standard WGTT assessment, the x-ray radiopaque marker (ROM) test, gives poor definition of colonic anatomy and the radiation dose required is undesirable in children. Our objective was to develop an alternative magnetic resonance imaging (MRI) WGTT measure to the x-ray ROM test and to demonstrate its initial feasibility in paediatric constipation.

**Methods:**

With the Nottingham Young Person's Advisory Group we developed a small (8 × 4 mm), inert polypropylene capsule shell filled with MRI-visible fat emulsion. The capsule can be imaged using MRI fat and water in-phase and out-of-phase imaging. Sixteen patients with constipation and 19 healthy participants aged 7 to 18 years old were recruited. Following a common ROM protocol, the participants swallowed 24 mini-capsules each day for 3 days and were imaged on days 4 and 7 using MRI. The number of successful studies (feasibility) and WGTT were assessed. Participants’ EuroQoL Visual Analogue Scale were also collected and compared between the day before the taking the first set of mini-capsules to the day after the last MRI study day.

**Results:**

The mini-capsules were imaged successfully in the colon of all participants. The WGTT was 78 ± 35 hours (mean ± standard deviation) for patients, and 36 ± 16 hours, *P* < 0.0001 for healthy controls. Carrying out the procedures did not change the EuroQoL Visual Analogue Scale scores before and after the procedures.

**Conclusions:**

Magnetic Resonance Imaging in Paediatric Constipation was a first-in-child feasibility study of a new medical device to measure WGTT in paediatric constipation using MRI. The study showed that the new method is feasible and is well tolerated.

**What Is Known/What Is New**

**What Is Known**

- Current methods for assessing whole gut transit time include the traditional abdominal x-ray and radiopaque markers.
- X-ray radiopaque marker methods expose children and young people ionizing radiation in the range 0.03 to 0.11 mSv.
- X-ray radiopaque markers produce 2-dimensional radiographs in which the bowel and location of the radiopaque markers may be difficult to distinguish.

**What Is New**

- We developed a new, magnetic resonance imaging visible mini-capsule, specifically aimed at children and young adults.
- This first-in-child feasibility study showed that whole gut transit time can be measured in paediatric constipation using the new mini-capsule device in conjunction with magnetic resonance imaging.

Functional constipation in childhood is common, with estimated prevalence of 14% (1–5). The diagnosis is based on symptom and is defined according to the Rome IV diagnostic criteria (6–8) which for a child older than 4 years of age must include 2 or more of the following (7): 2 or fewer defecations in the toilet/week; at least 1 episode of faecal incontinence/week; history of retentive posturing or excessive volitional stool retention; history of painful or hard bowel movements; presence of a large faecal mass in the rectum; and history of large diameter stools that can obstruct the toilet. Managing this condition can be challenging. Whole gut transit time (WGTT) imaging studies have long been used (9) as an objective measure which can assist in stratifying patients and directing management. Gut transit studies have been reported to help characterize normal transit (10), anorectal retention (outlet obstruction) (11), nonretentive faecal incontinence (12), and slow transit constipation (11,13) and can be particularly useful when medical history and/or physical examination is unreliable (13).

Current methods include both gamma scintigraphy and x-ray radiopaque markers (ROMs). Both techniques involve exposure to ionizing radiations, which is undesirable in young persons (14,15). The effective exposure dose provided can vary considerably depending on isotopes and techniques. Radiolabelled meals in gamma scintigraphy can provide between 0.1 mSv (16,17) up to 1 to 4 mSv (18) and x-ray can provide between 0.03 and 0.11 mSv (19). Scintigraphy uses radiolabelled tracers to determine transit time. Lack of standardization (20) and limited availability make its use problematic. In the conventional Metcalf ROMs method (21), the patients ingest a number of small, inert plastic pellets on 3 consecutive days after which a series of abdominal radiographs are taken to assess the location of the markers inside the gastrointestinal (GI) tract, thereby determining WGTT. The ROMs method is more widely available than scintigraphy. It is, however, often difficult to determine the location of the ROMs accurately on an x-ray film due to the tortuous structure of the large bowel and the limitations of 2-dimensional x-ray projection imaging.

Magnetic resonance imaging (MRI) has already revolutionized diagnostics in many fields and is recently coming of age for the assessment of functional GI diseases (22). WGTT has been recently measured using adult-sized, MRI visible capsules in health and constipation but the size of the capsules makes them unsuitable for children (23–26).

This study aimed therefore at developing an MRI alternative to the x-ray ROMs, specifically targeted for the first time at children and young adults, and at demonstrating feasibility of the test in paediatric constipation.

## METHODS

### Study Participants

**FIGURE 1:**
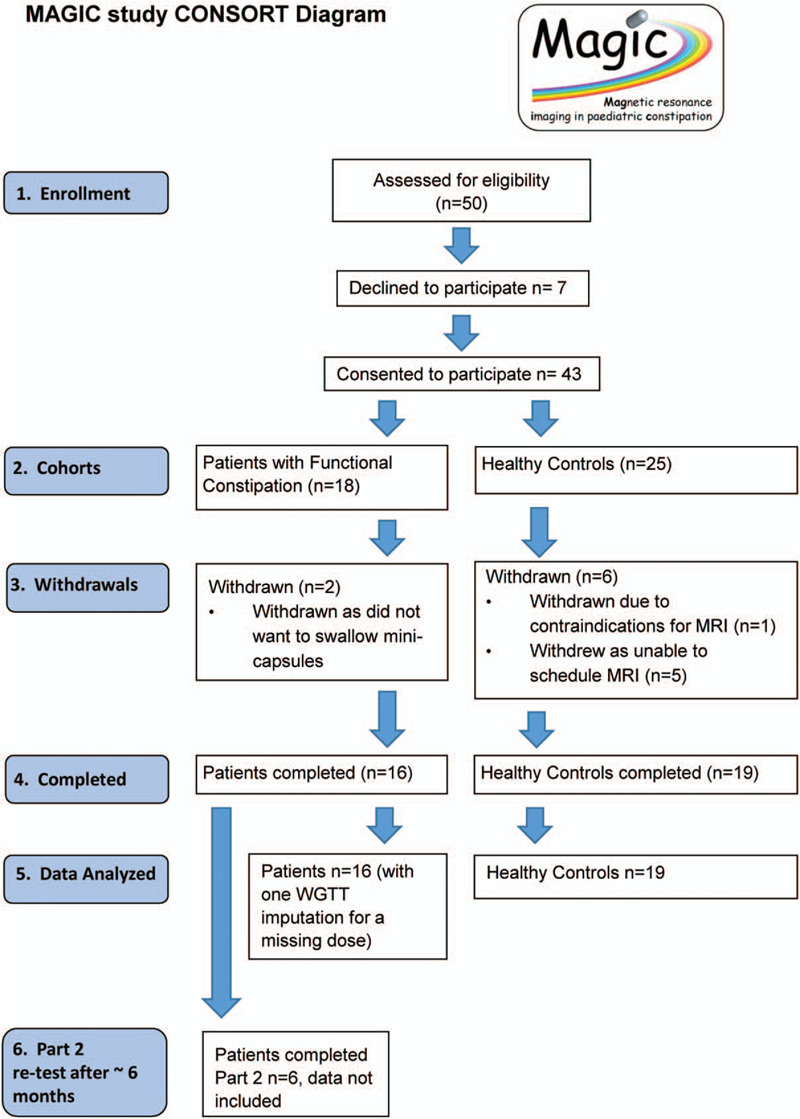
CONSORT diagram for the study. WGTT = whole gut transit time.

The Rome IV criteria was used to identify patients with childhood functional constipation following a referral either from primary or secondary care into a specialist clinic at Nottingham University Hospitals NHS Trust were recruited between April 2018 and June 2019 (Fig. 1). By the time they reached the specialist clinic, these patients were at various stages of treatment and there was no expectation that they would change treatment plan while participating as this was a feasibility study of the new methodology. During the same period, healthy participants, who had normal bowel habit and did not suffer from constipation or diarrhoea, were recruited by advertisement from the local community. The CONSORT diagram (27) is shown in Figure 1. All participants were aged between 7 and 18 years. A minimum age of 7 was selected as at this age participants are more likely to have had some experience of swallowing tablets. In participants younger than that age compliance with MRI procedures could also have been lower and motion artefacts could have been more frequent, precluding analysis. Participants with existing antegrade colonic enema procedure were excluded and patients with a history of GI surgery that could affect GI function, including colectomy or small bowel resection. Exclusion criteria included inability to lie flat and relatively still for <5 minutes and typical MRI scanning contraindications such as presence of metallic implants, pacemakers, and history of metallic foreign body in eyes. This study was approved by the UK National Research Ethics Committee (17/WM/0049), by the Medicines and Healthcare products Regulatory Agency (MHRA) (CI/2017/0054) and registered on Clinicaltrials.gov (NCT03564249).

### Study Design

This was an open label, feasibility, case-control study in a University setting. The intervention in this study mirrored a common x-ray ROM protocol (21). The rationale behind it is to load the GI tract with repeated daily doses of a plurality of markers to achieve a steady state and then image the location of the markers in the GI tract at predetermined time points (9). Many variants of this type of protocol have been proposed and we have chosen the one involving 3 daily doses of 24 markers each, which was also used for example for the common Sitzmarks ROMs product (9,28).

More specifically: participants were asked to swallow 24 mini-capsules each morning at home for 3 consecutive days (72 mini-capsules in total), by either mixing the mini-capsules in yoghurt or swallowing with water or fruit juice. The participants underwent a short MRI scan on day 4. If mini-capsules were still seen in the bowel, a second short MRI scan was performed on day 7. If mini-capsules were still seen in the bowel, they underwent a third MRI scan around day 26 to 28. This last scan was not part of the WGTT test but performed to collect data on possible retention of mini-capsules. EuroQoL Visual Analogue Scale (EQ-VAS) questionnaire data (29) were collected at baseline and each day of the study (day 0, before they started taking the mini-capsules to day 8). EQ-5D-Y data (30) were also collected at baseline. The questionnaire evaluates health-related life domains such as mobility, self-care, and pain. The lowest scores set possible for the EQ-5D-Y is 1,1,1,1,1 indicating no issues with any of the 5 domains. Scores higher than 1 in 1 or more domains indicate loss of quality of life (QoL). All questionnaires used with permission. In addition to the Consort Diagram at Figure 1, a CONSORT Checklist (27) was also used to standardize reporting; this is shown in Supplemental Digital Content 1 (Suppl CONSORT Checklist).

The patients who completed the feasibility study were to be invited to undergo an exact repeat of the protocol after approximately 6 months (part 2 of the study), to collect more pilot data and also start assessing potential changes in WGTT due to treatment. Due to project delays and funding timelines only 6 patients were able to come back and repeat the protocol. Although all 6 additional part 2 studies were completed safely, the numbers are too small for meaningful paired comparison and they are not included in this analysis or the results section.

### Study Objectives and Endpoints

The primary objective of this study was to develop the new device and technique and to assess its feasibility on paediatric constipation. The secondary objectives were to describe WGTT of controls and patients; to describe the safety of the technique; and to describe the effect of the technique on QoL.

The primary endpoint for feasibility was the number of successful measurements. The secondary endpoints were WGTT of controls and patients, the number of adverse events (AEs) for all participants, the change in EQ-VAS scores (young people health on the day) (30), and the baseline EQ-5D-Y.

### Mini-capsules

**FIGURE 2:**
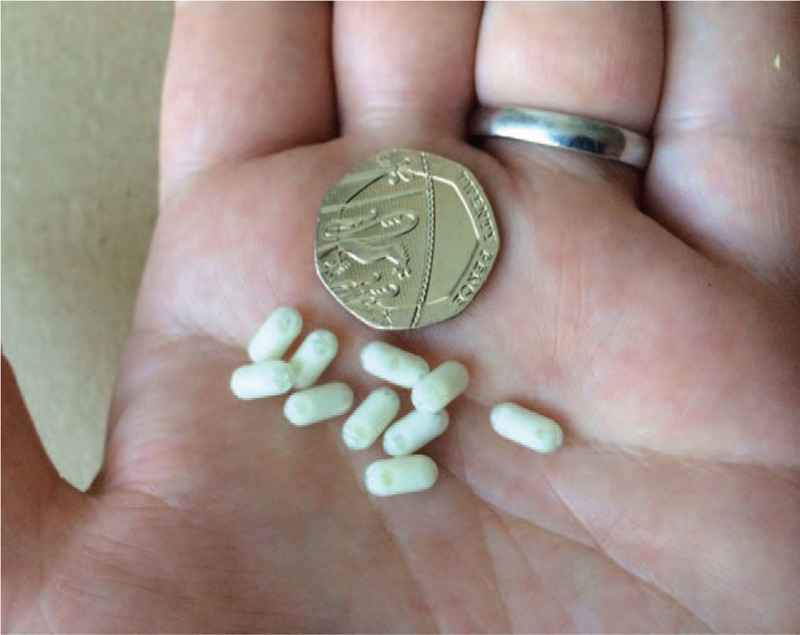
Mini-capsule MRI device markers pictured near a 20 pence coin (21 mm in diameter) for size comparison.

The mini-capsules are classed as an inert marker ingestible medical device (European Union Class IIa) designed in partnership with our Young Person Advisory Group (see Suppl Text on Supplemental Digital Content 2) and manufactured by JEB Technologies Limited (Hampstead Avenue, Mildenhall, UK). They enter the body via the oral cavity and travel inside the GI tract where they can be located using MRI (in good analogy with x-ray ROMs). The mini-capsules (Fig. 2) were made from medical grade polypropylene polymer (8 × 4 mm). The shell is invisible to MRI thus the mini-capsules were filled with an MRI-visible solution comprising oil and water, with trace amounts (1 μmol/L) of gadolinium (Gd) contrast agent for increased visibility, and the shell is designed to ensure that the contents are not released in the GI tract. The combination of fat, water, and Gd allowed us to exploit standard fat and water MRI sequences providing a unique, positive signal, MRI signature for the mini-capsules in the large bowel. At the time of the study, the mini-capsules were non-CE marked and not Food and Drug Administration approved. They have subsequently been trademarked as TransiCap.

### Magnetic Resonance Imaging

A 3 T, wide-bore Ingenia scanner (Philips, Best, The Netherlands) was used to acquire MRI. For the MRI the participants were not sedated and lay feet first on the scanner bed, in the supine position. A 3-dimensional 2-echo, mDIXON scan was then acquired to locate the mini-capsules. This provided 4 image types for each slice acquired: water only, fat only, fat and water in-phase, and out-of-phase images (31). The liquid filling of the mini-capsules was optimized in conjunction with the imaging sequence parameters so that on a derived subtraction of the out-of-phase image from the in-phase image the signal of the mini-capsules would be maximized compared to GI chime, intestinal water, and surrounding organs, facilitating their detection. The sequence was acquired both in coronal and axial planes, breaking down the 3D volume into stacks of short breath-holds to minimize respiratory motion. Five breath holds of 12.3 seconds each were required to collect the axial image set and 6 breath holds of 13.5 seconds were required for the coronal image set. The whole MRI procedure took approximately 15 to 20 minutes. The MRI sequence parameters including length in seconds are provided in Supplemental Digital Content 3 (Suppl Table 1).

Counting of the mini-capsules was performed preferentially on the coronal plane views because the anatomy of the colon is simpler to follow in that orientation. If the coronal images were blurred by motion or partly obscured by a very full bladder, then the axial images were of additional value for the counting. These were observations made at postprocessing stage. Four participants had some data sets that were more difficult to read because of blurring induced by respiratory motion caused by poor breath-holding during some of the imaging image acquisitions but possibly also by GI motility due to a recent meal. When blurring was noted by the radiographer at the time of scanning some image sets could be repeated immediately, which only required a few additional breath-holds. Acquiring both axial and coronal data sets and in separate stacks further helped because if one set was found to be blurry a different one could be analyzed. Analysis of 1 data set took approximately 10 minutes.

### Data Analysis

After each scan, the mini-capsules remaining in the gut were located and counted on the derived in-phase minus out-of-phase images and the WGTT calculated following the Sitzmarks ROM method (21). The calculation of WGTT assumes that that by loading the gut with repeated ingestion of the mini-capsules over 3 days, a steady state is reached so that: WGTT in hours = (72 hours/the number of mini-capsules ingested over 3 days) × the total number of mini-capsules remaining in the colon. The total number of mini-capsules ingested in this study over 3 days, 72, simplifies the multiplying factor so that WGTT = the total number of mini-capsules remaining in the colon at say 4. When some mini-capsules are remaining at day 7, their contribution to the total transit time is simply added to the count at day 4 using the same method. The principle of the WGTT marker method relies on loading of the bowel with a plurality of markers not necessarily on their specific total number (9); hence, the formula above can be adapted in case some of the mini-capsules were mistakenly not ingested, by simply changing the denominator for “the number of mini-capsules ingested over 3 days.” This happened in one instance in this study leading to one data imputation. All image data were analysed by a researcher with MRI radiographer background. The feasibility study was open label and they were not blind to which group the patients or controls belonged to, but they did not have knowledge of the individual patient histories. All statistical analyses were carried out using Prism version 6.07 (Graph Pad Software Inc, La Jolla, CA). Normality of the data was assessed using D’Agostino and Pearson test. The comparison of WGTT and EQ-VAS between patients and controls was carried out post hoc. The WGTT data were normally distributed and differences in WGTT between the patients and controls were compared using 2-tailed, unpaired *t* test. The EQ-VAS was compared between the baseline time point before the start of the study procedures and the day after the study procedures were completed (day 8). Data were considered significantly different at *P* < 0.05. There was no prior data on the new mini-capsules to estimate sample size and 25 controls as an acceptable sample size for a feasibility study (32,33) and allowing for dropouts.

## RESULTS

Thirty-five young persons were studied (Fig. 1). These comprised 16 patients (7 boys; 9 girls; 11 ± 3 years old; body mass index 25 ± 9 kg/m^2^) and 19 healthy young controls (8 boys; 11 girls; 16 ± 2 years old; body mass index 25 ± 5 kg/m^2^). The age between the 2 groups had a modest but significant difference (Mann Whitney *P* < 0.001) The individual participants’ characteristics are provided in Supplemental Digital Content 4 (Suppl Table 2).

### Number of Successful Measurements and Feasibility

All the 35 participants who ingested the mini-capsules completed the study. Only one of the subjects forgot to ingest one of the daily doses and the WGTT calculation was corrected for this. This showed very good acceptability of the mini-capsules and MRI procedures. Feedback from the participants and the parents, albeit not formally recorded, was positive.

The mini-capsules were imaged successfully in the colon of all participants using MRI.

**FIGURE 3:**
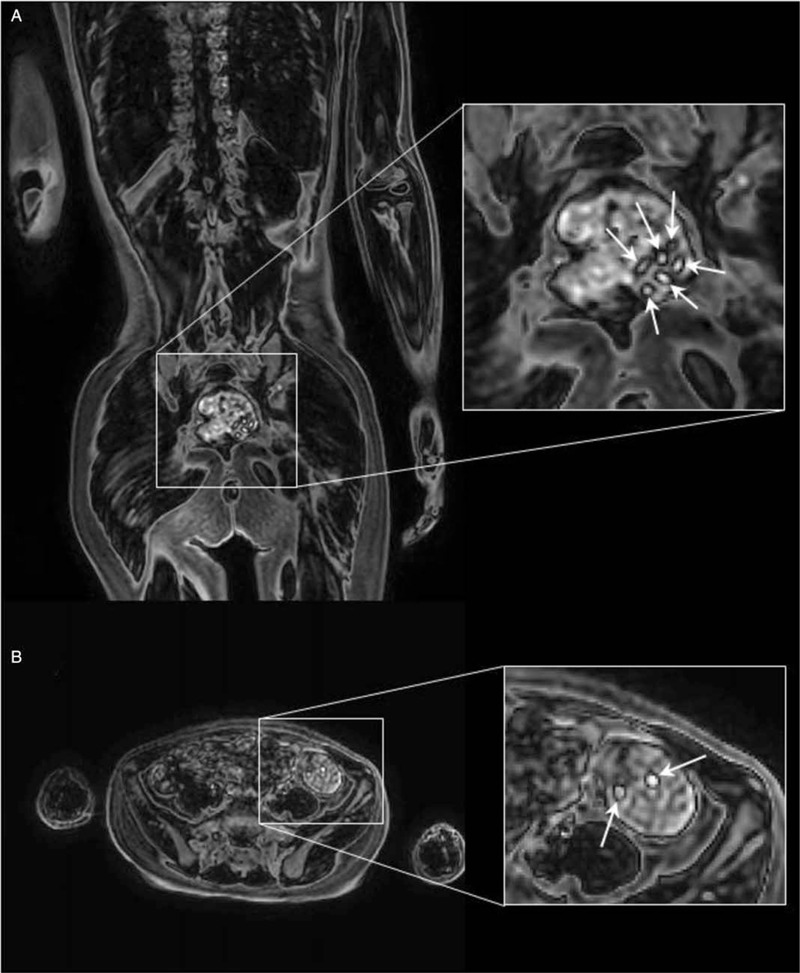
A, A coronal, derived in-phase minus out-of-phase magnetic resonance imaging (MRI) image of a 7-year-old patient participant, showing in the sigmoid/rectum 6 mini-capsules with positive signal against the chime. The mini-capsules are indicated by the white arrows in the corresponding expanded area on the right hand side. B, An axial, derived in-phase minus out-of-phase MRI image of the same patient participant, showing in the descending colon 2 mini-capsules with positive signal against the chyme. The mini-capsules are indicated by the white arrows in the corresponding expanded area on the right hand side.

Figure 3A shows the image of a single coronal image slice clearly showing the mini-capsules with bright, positive signal against the darker colon contents of a participant. The image shown in Figure 3B is an axial view from the same patient participant.

### Whole Gut Transit Time

**FIGURE 4:**
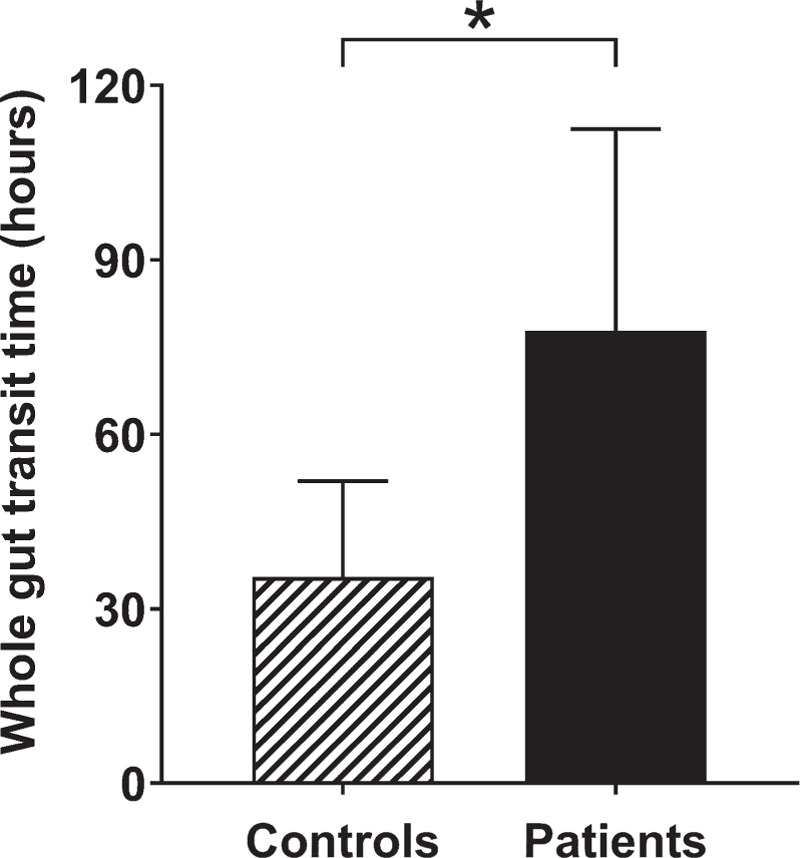
Whole gut transit time (mean and standard deviation) for young patients with constipation (n = 16) and healthy controls (n = 19). ^∗^*P* < 0.0001.

From the MRI images, it was possible to count the mini-capsules in the GI tract of all the participants. The WGTT (Fig. 4), calculated from the mini-capsules count, was 78 ± 35 hours for the young patients with constipation, significantly longer than that for the healthy controls 36 ± 16 hours (*P* < 0.0001).

### Number of Adverse Events and Safety

There were no serious adverse events in the study. There was only 1 AE in the study. This was a single episode of vomiting. It was categorized as mild and quickly resolved, requiring no follow-up and possibly related to the study procedures.

The number of mini-capsules detected in the gut decreased with time for all participants. In the patient group, the number significantly decreased from 57 ± 11 mini-capsules at day 4 to 19 ± 26 mini-capsules at day 7, *P* = 0.02. For the healthy controls the count significantly decreased from 31 ± 13 at day 4 to 4 ± 6 at day 7, *P* < 0.0001.

Some mini-capsules were present at day 7 in n = 11 patients and n = 9 healthy controls. These participants were recalled for a final scan at day 26 to 28 postingestion. No mini-capsules were detected in any of the participants at this final time point, thus in part confirming the European Union Class IIa classification of the device.

### EuroQoL Visual Analogue Scale Scores

There was no significant difference in EQ-VAS visual analogue scores provided by all participants pooled together at baseline before any procedure (87 ± 13) and after completing the mini-capsules and MRI intervention at day 8 (85 ± 18), Wilcoxon *P* = 0.79.

The young patients’ EQ-VAS grand mean was about 10% lower than for healthy controls with no significant differences between groups (*P* = 0.78). There are also no significant differences in EQ-VAS scores before and after the mini-capsules and MRI test for patients (Wilcoxon *P* = 0.54) and for healthy controls (Wilcoxon *P* = 0.55). A breakdown of data by patient group is shown in Supplemental Digital Content 5 (Suppl Fig. 1).

### EQ-5D-Y

All healthy controls scored their QoL at the lowest score 1 for all dimensions on the EQ-5D-Y questionnaires. Four patients indicated a loss of QoL in the questionnaires. A clerical error prevented the administration of the second batch of questionnaires after the MRI study procedures; therefore, those data are not available.

## DISCUSSION

The Magnetic Resonance Imaging in Paediatric Constipation study was a “first-in-child” clinical investigation of a novel medical device. As such we set out to assess feasibility, safety, and performance of using the new device in the intended population, for the intended purpose. The study was successful on various points.

Assessing feasibility of the new technique was the primary objective of the study. The data showed that using the new device and the MRI methods were feasible and the procedures acceptable with 35 paediatric participants ingesting the mini-capsules and undergoing MRI without refusal and completing the study. The intended method to image the mini-capsules, using a derived in-phase minus out-of-phase fat and water MRI images worked well. The mini-capsules appeared distinctively, with a high positive contrast against the colonic chyme.

Another strength of the whole project was the active participation of children and young persons in the codesign of the mini-capsules and of the feasibility study.

Having imaged the mini-capsules in the colon of these participants we have been able to count them, which provided a successful measure of WGTT using the simple algorithm described in the methods. Therefore, the new device met the intended purpose of being imaged and identified in the gut, monitoring gut transit time using MRI and of measuring the intended WGTT end point of the study in paediatric constipated participants and healthy controls. We have also collected a reference mean and a range of WGTT for the healthy controls, which was a secondary objective of the study. The new data on mean and standard deviation of WGTT for a reasonable number of paediatric patients and controls will also allow us initial inferences on sample size power calculations for future paediatric intervention studies.

When comparing our transit time results with other paediatric data in the literature, Gutierrez et al (34) found transit time in a constipated group of 49.6 versus 29.1 hours in a control nonconstipated group. They used an ROM method involving ingestion of 10 markers per day for 6 days followed by an x-ray on day 7. Zaslavsky et al (35) similarly found a transit time of 54.3 hours in an adolescent constipated group versus 30.2 hours in a control nonconstipated group, a delay in transit time of 40% compared to controls. They used 20 ROMs ingested each day for 3 days followed by an x-ray on day 4. The data collected here with our new mini-capsules showed a highly significant difference of 54% in WGTT between our young patients with constipation and healthy controls. This further corroborates evidence that the new device was able to detect a clinically significant difference between paediatric constipation and healthy controls.

In this study we measured WGTT by counting the number of capsules remaining in the bowel at predetermined time points. Measuring segmental transit times in different tract of the colon was not an outcome for this study. Segmental transit times were once more popular particularly to inform segmental surgical resection but recently controversy has been reported about this treatment and its uncertain benefits (36,37). The cross-sectional quality of MRI can provide an advantage over x-ray to assign the location of markers as bowel loops can overlap in the 2-dimensional x-ray abdominal film, though this needs to be assessed formally.

The study had a very good safety record. There was only 1 episode of vomit categorized as an AE. It is worth noting that this participant continued the study, completed the rest of the procedures, and agreed to come back for 1 of the part 2 studies which they completed successfully, further illustrating the mild nature of that AE.

The EQ-VAS scores showed no difference before and after the study procedures indicating that undergoing the study and the procedures did not alter the score of the patients and of the healthy participants.

The study had some limitations. The baseline EQ-5D-Y for the participants was not repeated after the study due to a clerical error thus preventing an appropriate assessment of changes in QoL.

The patient participants reached the specialist clinics in the hospital as they required expertise more than that available within primary and secondary care. Our pool included also those referred from other community paediatric clinics and general paediatric clinics within the region. As such, they were at various stages of treatment, when they participated in the study. There was no expectation that patients would change treatment plan whilst being investigated. It is interesting that despite this and their heterogeneity we demonstrated a longer WGTT in the patient group. At the same time this did not allow us to collect meaningful data on changes in gut transit upon treatment. The original design included an invitation to all patients to come back to repeat the feasibility study as a “part 2” approximately 6 months after the first participation. Due to delays in recruitment and funding timelines, and also some difficulty in communicating with the satellite district hospitals and practitioners, we were able to retest only 6 patients. They all repeated the MRI study safely, but the sample is too small to provide meaningful comparisons and these repeated data were excluded from the analysis. There was a significant difference in age between the 2 groups, although this was modest and did not seem to affect this feasibility study.

Some of the youngest participants reported feeling nervous at the start of the study, which is understandable for people who had not previously participated in a clinical investigation or had an MRI scan, but this did not stop any of them participating and they quickly relaxed and came back very happily for all the other visits. A further limitation of this study is that we did not set out to collect formal questionnaire feedback on how the procedures were perceived, the burden of attending and of going in the MRI scanner. A few data sets were blurred by motion. An observation made at postprocessing was that the large region of high intensity signal from a full bladder may make mini-capsule detection more difficult, particularly in the distal colon/rectum area, on the coronal image plane orientation, although further postprocessing techniques could overcome some difficulties. We also noted that participants who came in having just recently eaten a large breakfast or meal showed higher stomach and small bowel motility, which can also blur some image sets, particularly in the upper abdomen. Possible suggestions for improvement for the future could include more detailed explanations of the breath-holding and training young participants to hold their breath before the MRI scans, and recommending emptying of the bladder before going in the MRI scanner and a couple of hours of fasting before the MRI test. Clearly the utility of this mode of investigation is one to evaluate further. Constipation is a common problem and while the vast majority of children and young persons will respond to simple treatment with stool softeners and bowel stimulant, there is a significant proportion of patients who do not show signs of improvement despite follow-up. It is important to in these cases to evaluate the WGTT in order then to individualize therapy.

A noncontrast paediatric MRI scan to a single area of the body cost more than an x-ray (in the UK health system about 4 times more) so MRI cost and availability are issues to be taken into account. In the paediatric population, avoiding ionizing radiation exposure is a definite advantage of the new technique that should counterbalance the economic argument. The additional cross-sectional imaging additional information on the bowel anatomy that can be gathered within the same scan is also an advantage of MRI. In terms of availability, it is true that MRI units are particularly busy with clinical routine. When new bowel imaging scans, however, prove their worth they enter that clinical routine as it happened for MRI cholangiopancreatography and MRI enterography.

Here the primary aim of the study was simply to evaluate the feasibility of this test in children being looked after in a typical clinic set up. A full health economics evaluation needs to follow to evaluate the cost benefit analysis, taking into account not only the total duration of treatment and continued follow-up within a paediatric clinic but also school days lost because of faecal incontinence, and the impact of productivity for carers as they take time off to look after children with chronic constipation with overflow. The x-ray ROM protocol that we have copied for this initial study (3 daily doses of 24 markers) is a common one but it is not necessarily the only one used in past literature. The new MRI mini-capsules could be used in the future with other imaging protocols such as a single dose, single imaging time point (9) and also in adults, although further investigation and validation will be needed. A new multicentre study of the mini-capsules in paediatric constipation was funded and is under way (Trial ID: ISRCTN42273449).

## CONCLUSIONS

Magnetic Resonance Imaging in Paediatric Constipation was a first-in-child feasibility study of the new mini-capsule medical device (TransiCap) to measure WGTT in paediatric constipation using MRI. The study showed that the device met safety and performance objectives as per the intended purpose, with excellent feasibility and safety of using the new device in conjunction with MRI. The new device may represent a modern alternative to current x-ray ROM methods while not exposing the young patients to any ionizing radiation and at the same time providing high-quality cross-sectional images of the bowel. The clinical efficacy of using the mini-capsule test in clinical practice remains to be determined.

## Supplementary Material

Supplemental Digital Content

## Supplementary Material

Supplemental Digital Content

## Supplementary Material

Supplemental Digital Content

## Supplementary Material

Supplemental Digital Content

## Supplementary Material

Supplemental Digital Content
