# Supplementary material for: Feasibility Study of a New Magnetic Resonance Imaging Mini-capsule Device to Measure Whole Gut Transit Time in Paediatric Constipation
Source: J Pediatr Gastroenterol Nutr. 2020 Aug 17;71(5):604–11. doi: 10.1097/MPG.0000000000002910 (PMC7575025; doi:10.1097/MPG.0000000000002910)

**
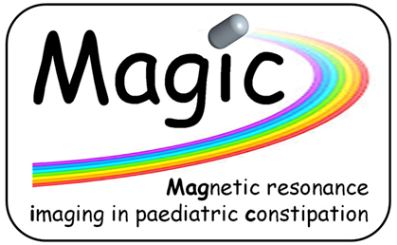
Supplemental Digital Content 2: Supplemental Text**

**Patient and Public Involvement and Engagement**

The patient and public involvement and engagement (PPI/E) in this project was recurrent and meaningful since inception. We co-designed and co-produced the Transicap^TM^ mini-capsules and the packaging, with the Young Person Advisory Group (YPAG) working in person with the designers at Renfrew Group International (Abbey Meadows, Leicester, UK) and the technology consultants from the NIHR from the Enteric Health Technology Cooperative. Together with the YPAG members we designed the MAGIC project website [www.gastrointestinalmri.org.uk](http://www.gastrointestinalmri.org.uk), we scripted, produced and narrated two novel, animated pediatric video info sheets, now Ethics approved and on YouTube <https://youtu.be/luvIutiTvr4> and <https://youtu.be/w5O8lhZqEs8>.


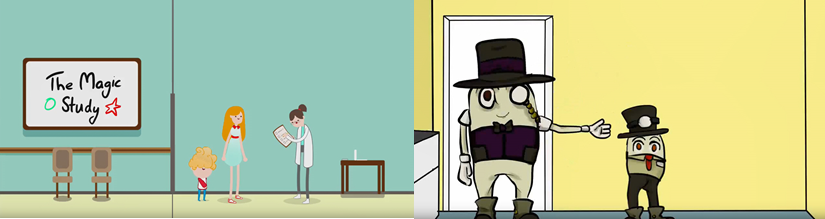


We wrote age-appropriate Ethics information sheets (praised by the Ethics Committee) and presented together at the podium of the Nottingham Pediatric Research Showcase 2018 (winning 2^nd^ best oral presentation) and at the UK Clinical Research Facilities conference 2019.

We continue working with the YPAG and some of their comments on the progress of the co-production can be seen on this video edited from a round-table discussion about the YPAG’s participation in the MAGIC programme <https://www.youtube.com/watch?v=tPRt75xdq2k>.


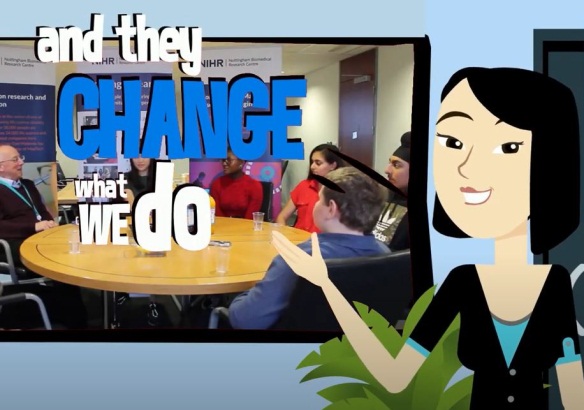

Supplement: Supplemental Digital Content [file jpga-71-604-s002.docx]
