# Supplementary material for: Feasibility Study of a New Magnetic Resonance Imaging Mini-capsule Device to Measure Whole Gut Transit Time in Paediatric Constipation
Source: J Pediatr Gastroenterol Nutr. 2020 Aug 17;71(5):604–11. doi: 10.1097/MPG.0000000000002910 (PMC7575025; doi:10.1097/MPG.0000000000002910)
Supplement: Supplemental Digital Content [file jpga-71-604-s003.docx]

**Supplemental Digital Content 3: Table 1**

| SUPPLEMENTAL TABLE 1. MRI sequence parameters | |  |
| --- | --- | --- |
| MRI sequence | 3D T1 weighted TFE ^*^ | |
| Image orientation | Axial | Coronal |
| Field of View | 350 mm (RL) × 280 mm (AP) | 348 mm (RL) × 250 mm (HF) |
| Image stacks ^†^ | 5 ^‡^ | 6 ^§^ |
| Slices per stack | 33 | 27 |
| Length of breath hold per stack | 12.3 s | 13.5 s |
| SENSE acceleration factor | 2 | 2 |
| Signal averaging | 1 | 1 |
| Flip angle | 20° | 20° |
| Repetition time TR | 10 ms | 10 ms |
| Echo times TE_1_ / TE_2_ | 1.32ms / 2.2 ms | 1.32ms / 2.2 ms |
| Reconstruction matrix | 400 × 400 | 400 × 400 |
| Acquired image resolution | 1.8 mm × 1.8 mm × 4.4 mm | 1.8 mm × 1.8 mm × 4.4 mm |
| Reconstructed image resolution | 0.88 mm × 0.88 mm × 2.2 mm | 0.87 mm × 0.87 mm × 2.2 mm |

^*^ mDIXON sequence on Philips MRI scanner used

^†^ The image stacks (packages) were acquired with no gaps between them, reconstructed independently and then put back together as a full section.

^‡^ The scanner table moved between each stack

^§^ The scanner table moved between a 'top' and a 'bottom' sections and then 3 stacks were acquired at each of these two table positions.
