# Supplementary material for: Feasibility Study of a New Magnetic Resonance Imaging Mini-capsule Device to Measure Whole Gut Transit Time in Paediatric Constipation
Source: J Pediatr Gastroenterol Nutr. 2020 Aug 17;71(5):604–11. doi: 10.1097/MPG.0000000000002910 (PMC7575025; doi:10.1097/MPG.0000000000002910)
Supplement: Supplemental Digital Content [file jpga-71-604-s004.docx]

**Supplemental Digital Content 4: Table 2**

| **SUPPLEMENTAL TABLE 2. Individual participants’ characteristics** | | | | | | |
| --- | --- | --- | --- | --- | --- | --- |
| Participant number | Patient or healthy control | Age | Gender | Weight (kg) | Height (m) | BMI (kg/m^2^) |
| 1 | Healthy control | 16 | Female | 63 | 1.65 | 23.0 |
| 2 | Healthy control | 17 | Female | 65 | 1.63 | 24.5 |
| 3 | Patient | 8 | Male | 60 | 1.30 | 35.5 |
| 4 | Healthy control | 11 | Male | 48 | 1.60 | 18.7 |
| 5 | Healthy control | 14 | Male | 52 | 1.60 | 20.3 |
| 6 | Healthy control | 16 | Female | 63 | 1.70 | 21.8 |
| 7 | Patient | 11 | Male | 99 | 1.67 | 35.5 |
| 8 | Healthy control | 17 | Male | 72 | 1.79 | 22.5 |
| 9 | Healthy control | 17 | Female | 70 | 1.70 | 24.2 |
| 10 | Healthy control | 15 | Female | 58 | 1.59 | 22.8 |
| 11 | Patient | 10 | Female | 41 | 1.05 | 37.1 |
| 12 | Healthy control | 18 | Male | 85 | 1.69 | 29.8 |
| 13 | Healthy control | 18 | Male | 91 | 1.75 | 29.7 |
| 14 | Healthy control | 17 | Male | 70 | 1.73 | 23.4 |
| 15 | Healthy control | 18 | Male | 106 | 1.67 | 38.0 |
| 16 | Healthy control | 16 | Female | 59 | 1.71 | 20.2 |
| 17 | Patient | 13 | Female | 60 | 1.64 | 22.3 |
| 18 | Patient | 7 | Male | 25 | 1.28 | 15.5 |
| 19 | Healthy control | 14 | Female | 55 | 1.60 | 21.4 |
| 20 | Patient | 8 | Female | 48 | 1.30 | 28.4 |
| 21 | Healthy control | 17 | Female | 57 | 1.52 | 24.7 |
| 22 | Healthy control | 17 | Male | 80 | 1.85 | 23.4 |
| 23 | Healthy control | 15 | Female | 97 | 1.79 | 30.3 |
| 24 | Healthy control | 18 | Female | 57 | 1.63 | 21.5 |
| 25 | Patient | 10 | Male | 34 | 1.20 | 23.6 |
| 26 | Patient | 16 | Female | 68 | 1.55 | 28.3 |
| 27 | Patient | 10 | Female | 22 | 1.28 | 13.2 |
| 28 | Patient | 12 | Male | 34 | 1.40 | 17.3 |
| 29 | Patient | 9 | Female | 35 | 1.20 | 24.3 |
| 30 | Patient | 18 | Female | 52 | 1.25 | 33.3 |
| 31 | Patient | 13 | Male | 60 | 1.54 | 25.3 |
| 32 | Patient | 13 | Male | 42 | 1.54 | 17.7 |
| 33 | Patient | 14 | Female | 63 | 1.64 | 23.4 |
| 34 | Patient | 7 | Female | 23 | 1.20 | 15.9 |
| 35 | Healthy control | 10 | Female | 42 | 1.45 | 20.0 |
