# Supplementary material for: Feasibility Study of a New Magnetic Resonance Imaging Mini-capsule Device to Measure Whole Gut Transit Time in Paediatric Constipation
Source: J Pediatr Gastroenterol Nutr. 2020 Aug 17;71(5):604–11. doi: 10.1097/MPG.0000000000002910 (PMC7575025; doi:10.1097/MPG.0000000000002910)

**Supplemental Digital Content 5 – Suppl Figure 1.** Young participants’ EQ-VAS scores before and after undergoing the mini-capsules ingestion and MRI scan procedures. The values shown are mean±standard deviation. Wilcoxon’s *P* = 0.54 the patients and *P* = 0.55 for the healthy controls comparing before and after the study procedures respectively.


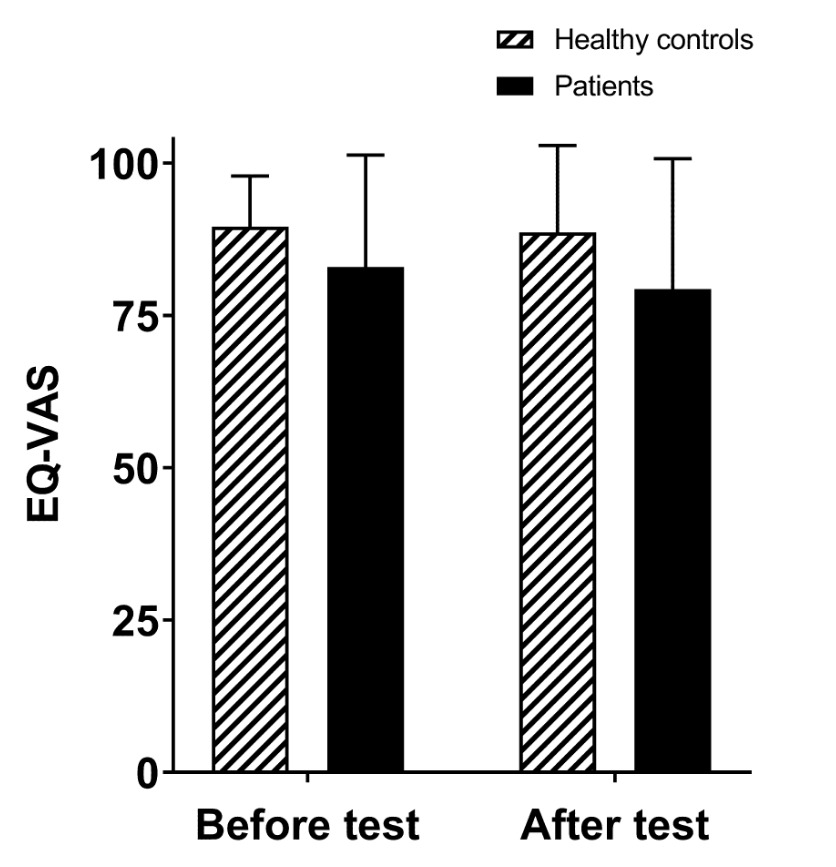

Supplement: Supplemental Digital Content [file jpga-71-604-s005.doc]
